# The complete mitochondrial genome of the gray garden slug *Deroceras reticulatum* (Gastropoda: Pulmonata: Stylommatophora)

**DOI:** 10.1080/23802359.2017.1318677

**Published:** 2017-04-27

**Authors:** Seung-Joon Ahn, Ruth Martin, Sujaya Rao, Man-Yeon Choi

**Affiliations:** a USDA-ARS Horticultural Crops Research Unit, Corvallis, OR, USA;; b Department of Crop and Soil Science, Oregon State University, Corvallis, OR, USA;; c USDA-ARS Forage Seed and Cereal Research Unit, Corvallis, OR, USA

**Keywords:** *Deroceras reticulatum*, gray garden slug, mitochondrial genome

## Abstract

The complete mitochondrial genome sequence of *Deroceras reticulatum* has been sequenced and annotated in this study. The mitogenome of *D. reticulatum* is 14,048 base pairs in length, and contains 13 protein-coding genes (PCGs), 22 transfer RNA genes, and 2 ribosomal RNA genes. The overall base composition is 31.0% A, 12.2% C, 17.7% G, and 39.1% T. Based on phylogenetic analysis using the amino acid sequences of PCGs, *D. reticulatum* was shown to be closely related to other species of Stylommatophora. The first mitochondrial genome from the Agrolimacidae family provides valuable molecular data for taxonomical identification and further evolutionary studies of terrestrial slugs.

The gray garden slug, *Deroceras reticulatum* (Gastropoda: Pulmonata: Stylommatophora), is a common terrestrial mollusc native in Europe with global distribution (Godan 1983; Tulli et al. 2009), which damages a wide range of vegetables and field crops (Port and Port 1989; South 1992). However, virtually nothing is known about genetic information of the species. In this study, we determined the complete mitochondrial genome of *D. reticulatum*.

A specimen of *D. reticulatum* was collected from a rural area in Corvallis, Oregon, USA. After morphological identification, the specimen was kept at –80 °C in the USDA-ARS laboratory under the accession number DR151201. Partial mitochondrial sequences were obtained from the whole body transcriptome generated by Illumina HiSeq 2000 and assembled by Trinity *de novo* assembly. The entire mitochondrial genome sequence was confirmed by PCR using specific primers and resequencing. Sequence annotations were performed by MITOS (http://mitos.bioinf.uni-leipzig.de; Bernt et al. 2013) and ARWEN 1.2 (http://mbio-serv2.mbioekol.lu.se/ARWEN/; Laslett and Canbäck 2008), followed by manual validation of the coding regions using the ORFfinder tool (https://www.ncbi.nlm.nih.gov/orffinder/) and comparing other molluscan mitochondrial genomes.

The complete circular mitochondrial genome of *D. reticulatum* is 14,048 bp in length, consisting of 13 protein-coding genes (PCGs), 22 transfer RNA (tRNA) genes, and 2 ribosomal RNA (rRNA) genes (GenBank accession number: KY765589). The overall base composition was 31.0% A, 12.2% C, 17.7% G and 39.1% T, indicating a very high AT content (70.1%). The start codon ATG was used in five PCGs (COX2, ATP8, ATP6, ND3, and ND4), ATA was used in four PCGs (ND6, ND5, ND1, and ND4L), and TTG was used in three PCGs (COX1, CYTB and ND2). Ten PCGs ended with the TAA stop codon, whereas the other three PCGs (CYTB, ATP6 and ND2) used TAG as stop codon. It is notable that the C-terminal sequence of COX1 had approximately an additional 50 aa when compared to other molluscan orthologues. The 22 predicted tRNA genes were spread over the whole mitochondrial genome and varied in length from 53 to 65 bp. The length of the two rRNA genes were 565 bp for the small rRNA (s-rRNA) and 871 bp for the large rRNA (l-rRNA). Comparative genome analysis revealed that the gene order and orientation of the *D. reticulatum* mitochondrial genome are similar to most other Pulmonata mitochondrial genomes.

To determine the phylogenetic relationship of *D. reticulatum* within Pulmonata, a maximum-likelihood tree was constructed by based on the concatenated amino acid sequences of all PCGs (except ATP8) from 20 different Pulmonata species including *D. reticulatum*. The sequence alignment and the tree construction were implemented in MEGA6.0 (Tamura et al. 2013). The results revealed that *D. reticulatum* is closely related to other species of Stylommatophora, but is in a different clade from Systellommatophora and Ellobioidea (Figure 1). The complete mitochondrial genome of *D. reticulatum* provides valuable molecular data for taxonomical identification and further evolutionary studies of terrestrial slugs.

**Figure 1.**
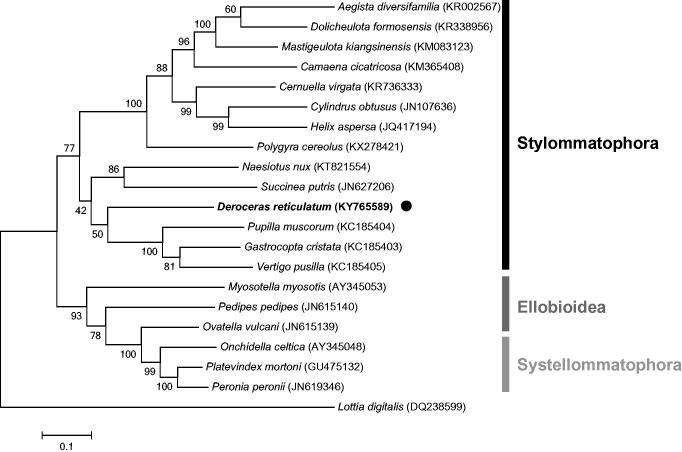
The phylogenetic tree of *Deroceras reticulatum* and related species in Pulmonata using amino acid sequences of the PCGs. The tree was constructed by the maximum-likelihood method with 1000 bootstrap replicates implemented in MEGA6.0 (Tamura et al. 2013). The *Lottia digitalis* (limpet) mitochondrial genome was used as an outgroup. Mitochondrial genome sequences were obtained from GenBank and their accession numbers are indicated next to species name.
